# A Retrospective Study on the Incidence of Kaposi Sarcoma in the United States From 1999 to 2020 Using the Centers for Disease Control and Prevention Wide-Ranging Online Data for Epidemiological Research (CDC WONDER) Database

**DOI:** 10.7759/cureus.77213

**Published:** 2025-01-10

**Authors:** Deepthi Balasubramanian, Sahana Srinivasan, Ponnu M Paul, Nahyun Ko, Shivani Garlapati

**Affiliations:** 1 Internal Medicine, Rajah Muthiah Medical College, Annamalai University, Chidambaram, IND; 2 General Medicine, Government Medical College, Omandurar Government Estate, Chennai, IND; 3 Medicine, Malankara Orthodox Syrian Church Medical College, Kolenchery, IND; 4 Medicine, Seoul National University College of Medicine, Seoul, PRK; 5 Internal Medicine, Vydehi Institute of Medical Sciences and Research Centre, Bangalore, IND

**Keywords:** cancer, cdc-wonder, disparity, incidence, kaposi sarcoma, prevention

## Abstract

Introduction

Studying cancer incidence is important to understand the cancer risk factors, tracking trends, planning resources and developing prevention and treatment methods. Examining the incidence of Kaposi sarcoma (KS) alongside different variables enables researchers to gain insights into the disease's underlying causes and risk factors. This understanding aids in developing more focused research and interventions.

Methodology

A retrospective study was conducted using the Centers for Disease Control and Prevention Wide-Ranging Online Data for Epidemiological Research (CDC WONDER) database. Data were extracted on July 31, 2024. Incidence of cancer was studied based on the following variables: age, gender, race and geographic location.

Results

This study describes the demographic characteristics of KS patients in the United States between 1999 and 2020 based on age, gender, and race. Based on age and gender, the crude rate per 1,00,000 was highest initially in ages 75 and above and the male gender. Based on race, the crude rate per 1,00,000 was highest in the Black or African American race. Based on the state, the incidence of KS was highest in California state, followed by New York, and based on the year, the incidence of KS was highest in 2000.

Conclusions

Incidence in the 35-44 years age group has shown a significant decreasing trend from 2001 to 2020. Other age groups have not shown an evident decreasing or increasing trend over the years. A decreasing trend was observed in White and Black or African American populations, and stable in Asian or Pacific Islander populations, and other races and unknown combined populations.

## Introduction

Kaposi sarcoma (KS), first described by the Hungarian dermatologist Moriz Kaposi in 1872, is a disease characterised by proliferative vascular lesions, caused by KS-associated herpesvirus (KSHV), also known as human herpesvirus 8 (HHV 8) [1]. Since the onset of the HIV epidemic, a new variant of KS has emerged, known as AIDS-associated KS (AIDS-KS), affecting individuals with HIV [1]. It is also seen in patients on long-term immunosuppressive treatment such as after transplantation. KS presents in various forms, from isolated cutaneous lesions to involvement of visceral organs like the mouth, gastrointestinal tract, or respiratory tract, often with overlapping features [2].

AIDS-KS is the most common type of KS in the United States [3]. The distribution of KSHV varies geographically, with reports documenting seroprevalences as high as 90% in some areas of sub-Saharan Africa and < 10% in the United States and most of Europe, even though the reasons are poorly understood [4]. A multiregional, multicohort study showed that the incidence of KS per 100,000 person-years in people living with HIV (PLWH) was 280 in South Africa, 244 in Latin America, 237 in North America, 180 in Europe, and 52 in Asia-Pacific [3]. A meta-analysis revealed that the prevalence of KS is higher in men than in women, likely due to various contributing factors like hormonal, viral, and genetic factors and high-risk behavior. KS exhibits significant morbidity and mortality and ranks globally as the most common HIV-related malignancy [5,6]

The incidence of KS in PLWH has dramatically decreased since the advent of antiretroviral therapy (ART) [7,8]. Radiotherapy is the preferred treatment for localized KS, as it effectively mages pain, controls bleeding, alleviates edema, and provides local control of skin and mucosal lesions. Meanwhile, pegylated liposomal doxorubicin is approved as the first-line chemotherapy regimen for AIDS-KS [9,10].

Studying KS incidence with factors like age, gender, race, and geographic location helps researchers understand the disease's underlying causes and risk factors, contributing to more targeted research and intervention. It also helps assess the effectiveness of treatments and informs public health policies. This study utilized the Centers for Disease Control Prevention Wide-Ranging Online Data for Epidemiologic Research (CDC WONDER) database to evaluate KS incidence and demographic variations trends [11]. The current lack of research in this area highlights the importance and need for this investigation. By addressing this gap, this study provides valuable data that can improve disease management, refine treatment strategies, and guide public health interventions more effectively.

This study aims to assess the incidence of KS in the United States from 1999 to 2020, based on demographic variables of age, gender, and race.

## Materials and methods

A retrospective original research study was conducted using data from the CDC-WONDER database (https://wonder.cdc.gov/). This publicly accessible database provides de-identified, aggregate-level data on various health outcomes, including mortality and cancer incidence, allowing for large-scale epidemiologic studies. As CDC-WONDER contains only de-identified data of a public nature, it qualifies as non-human participant research, and thus, no ethics committee approval was required for this study in accordance with relevant guidelines and standards for data privacy and ethical research practice.

All data was extracted on a single day, May 18, 2024, from the CDC-WONDER database, focusing on cancer incidence data spanning from 1999 to 2020. The inclusion criteria were defined as all reported cases of KS in the United States during this period. The variables selected for analysis included year of diagnosis (to assess temporal trends), age, sex, race (to analyze demographic trends), and data from all United States states to ensure geographic representation. The decision to focus on KS was based on its clinical significance and epidemiologic patterns, particularly within immunocompromised populations. Other types of cancer were excluded from the analysis to maintain the specificity of the study’s objectives, and cases not meeting the inclusion criteria were systematically excluded.

Once the data was extracted, it was exported to a Microsoft Excel spreadsheet (Microsoft Corporation, Redmond, Washington, United States) for initial organization and quality checks. Subsequently, statistical analysis was conducted using R version 4.3.1 (R Foundation for Statistical Computing, Vienna, Austria). This included descriptive statistics for demographic variables and trends in KS incidence over time. The R software was chosen for its flexibility and robust statistical packages that support epidemiological analyses. Graphical representation of the data was performed using GGPlot2, version 3.5.0 (Released 2024; https://www.tidyverse.org/blog/2024/02/ggplot2-3-5-0/), which allowed for the creation of clear and informative visualizations of the trends in incidence rates over time and across demographic categories. These figures were used to highlight key findings regarding the distribution and temporal changes in KS cases across various populations in the United States.

## Results

From 1999 to 2020, the incidence of KS was 27,791 in a total population of 6,722,531,044 in the CDC WONDER database. The crude rate per 100,00 was 0.4. Table 1 shows demographic characteristics of KS patients in United States from 1999-2020 based on age, gender and race. The crude rate per 100,000 was highest in the age group 75 years and above, male gender. Based on race, the crude rate per 100,000 was highest in the Black or African American race.

**Table 1:** Demographic characteristics of Kaposi sarcoma patients in the United States from 1999 to 2020 based on age, gender, and race.

Characteristics	Total Population	Count, n (%)	Crude rate per 100,000
Age			
15-24 years	932,609,300	723 (2.6%)	0.1
25-34 years	917,100,206	5058 (18.22%)	0.6
35-44 years	927,607,896	7560 (27.23%)	0.8
45-54 years	924,339,992	5040 (18.15%)	0.5
55-64 years	763,946,196	2575 (9.27%)	0.3
65-74 years	508,470,217	2257 (8.13%)	0.4
​​≥75 years​	416,497,118	4555 (16.4%)	1.1
Gender			
Male	3,305,492,262	24643 (88.67%)	0.7
Female	3,417,038,782	3148 (11.33%)	0.1
Race			
American Indian or Alaskan Native	88,290,475	172 (0.62%)	0.2
Asian or Pacific Islander	373,820,618	593 (2.13%)	0.2
Black or African American	914,358,967	7816 (28.12%)	0.9
White	5,346,060,984	18254 (65.68%)	0.3
Other races	Not Applicable	956 (3.44%)	Not Applicable

**Figure 1:**
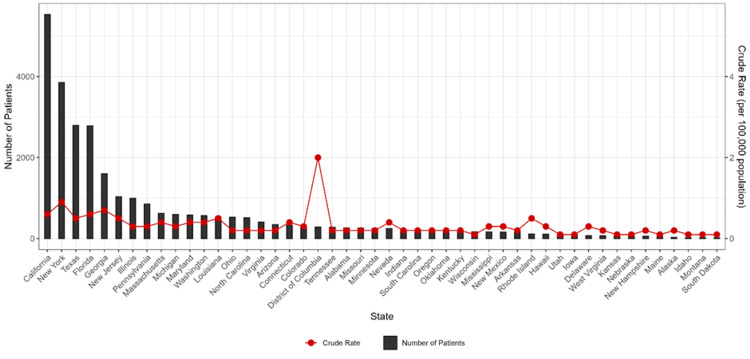
State-wise distribution of Kaposi sarcoma patients in the United States from 1999 to 2020.

Figure 1 shows state-wise distribution of KS patients in United States in 1999-2020. In the last 22 years, the incidence of KS was highest in California state, followed by New York and Texas. It was lowest in Idaho, Montana and South Dakota. The crude rate per 100,000 was highest in the District of Columbia.

**Figure 2:**
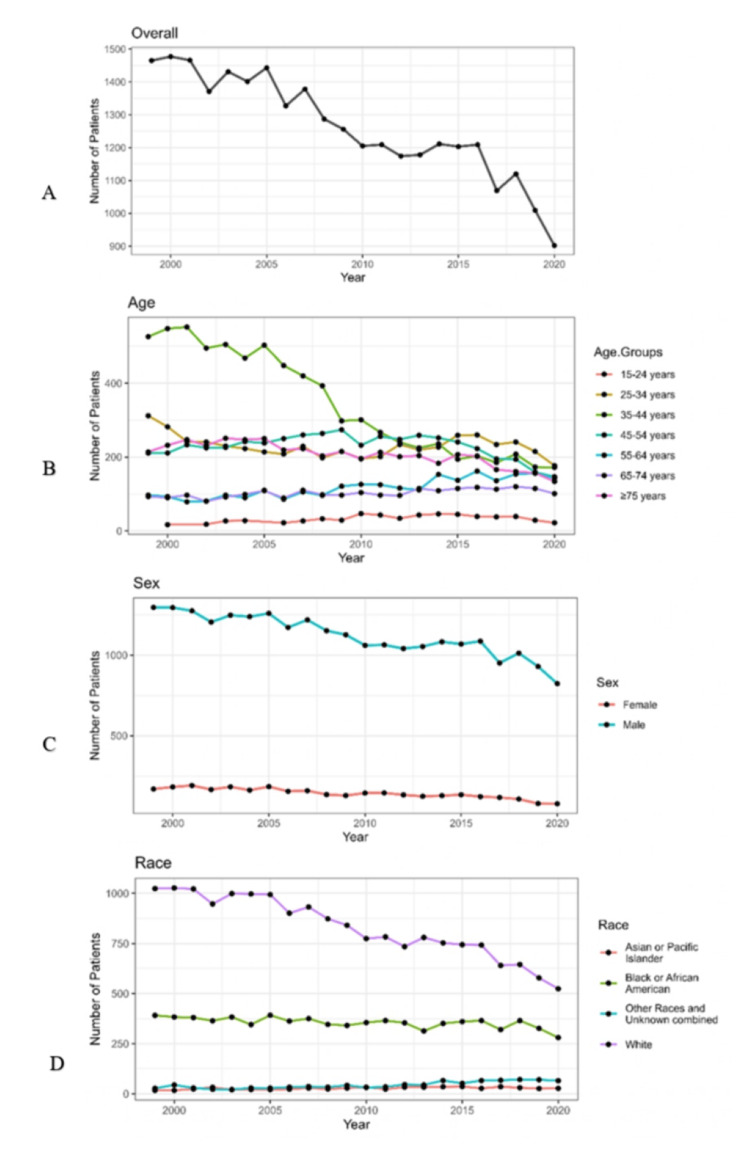
Temporal trends in the incidence of Kaposi sarcoma in the United States from 1999 to 2020 based on age, gender, and race. A. Overall temporal trends in the incidence of Kaposi sarcoma; B. Temporal trends in the incidence of Kaposi sarcoma based on age; C. Temporal trends in the incidence of Kaposi sarcoma based on sex; D. Temporal trends in the incidence of Kaposi sarcoma based on race

Figure 2A shows the temporal trends in the incidence of KS in United States from 1999 to 2020 based on age, gender, and race. The incidence of KS was highest in 2000, followed by 1999 and 2001. The incidence shows a decreasing trend over the years. The incidence shows a decreasing trend in the age group 35-44 years (Figure 2B). A mild rise in incidence is noticed in the 25-34 age group post 2014 and in the 55-64 age group after 2013. While the incidence remains stable in females, a decreasing trend in incidence is seen in the male population over the years (Figure 2C). Similarly, a decreasing trend is seen in case of the White population over the years, while that of the other races remain fairly constant (Figure 2D).

## Discussion

This study revealed that out of the 6,722,531,044 total population analyzed, the crude rate per 100,000 was 0.4 over 22 years. Incidence of KS was highest initially in ages 35-44, but after 2015 was higher in ages 25-34. The temporal trends show a falling trend with a steep fall after 2018 in the crude rate of KS. The crude rate per 100,000 patients aged over 75 years was 1.1, in male gender was 0.7, and in Black or African American was 0.9. The state-wise distribution of KS patients in the United States in 1999-2020 shows the incidence was highest in California, followed by New York. The incidence was lowest in Idaho, Montana, and South Dakota.

Studying the incidence of KS in population-wide trends globally and addressing the healthcare disparities and treatment plans might contribute to preventing KS [3]. KS presents in four clinical forms, all linked to HHV8 infection: classic, endemic, immunosuppression-related, and AIDS-KS [12]. KS is a rare cancer globally, but it is more common in certain countries in southern and eastern Africa and common in PLWH [3,13]. Studying the temporal trends over the years in KS is essential for improving cancer surveillance by providing estimates of cancer incidence on a broad scale. They enable effective planning and monitoring of evidence-based cancer control strategies across various healthcare levels [14], and continue to play an essential role in monitoring the burden of KS and improving its outcome [15].

A study in Europe shows there is an increased crude rate of KS in older people when compared to younger people. Still, there was an increased incidence of KS in younger people in 2003-2007, mainly AIDS-related, and an increased number of males were affected [15]. KS has always been more common in males than females [16]. HHV-8 infections show significant differences in geographic regions and ethnicity [17]. Recent studies also showed increased male incidence in Turkey, the Netherlands, and some areas of Africa [3,12,16]. In the United States, there is an increased incidence of KS in Black or African American populations compared to the White American population [18]. Overall, the incidence of AIDS-KS has dramatically decreased over the years with the introduction of ART [19].

Although the incidence of KS has decreased in the United States overall, the geographic and racial disparities in KS incidence and survival are still present [20]. Lifestyle factors may influence viral dynamics, immune function, and disease progression, but more studies are needed to fully understand these interactions, especially considering potential differences between males and females [16]. Since some ethnicities and geographical regions are more affected, it is vital to develop targeted public health interventions like targeting HIV prevention and treatment, optimizing treatment strategies, and improving patient outcomes [18]. Future studies are necessary to identify the specific risk factors contributing to the high incidence of KS among vulnerable populations, implement policies that encourage early screening and timely diagnosis, and establish effective preventive measures.

Limitations

This study has some limitations. The limitations of CDC WONDER database are the limitations of our study as well, like inclusion of only those cases that are available through the database. Additionally, the data collection process was halted during COVID-19 pandemic for the database and thus the latest data from 2021 to 2023 were not available on CDC WONDER and thus could not be included in this study. Details about the stage at diagnosis, grade at diagnosis, and outcome of KS could not be collected using this method, as it is not available on CDC WONDER database.

## Conclusions

This study provides a comprehensive analysis of KS incidence in the United States from 1999 to 2020, highlighting key demographic and geographic variations. Since temporal trends offer valuable insights into cancer incidence, this data can be used to shape policies focused on early identification and screening. By analyzing patterns over time, healthcare systems can target high-risk populations, optimize screening programs, and implement preventative measures, ultimately improving patient outcomes through early detection and intervention strategies. Further research should prioritize identifying risk factors, lifestyle influences, geographic and ethnic variations, as well as the male predominance of the disease. This can help better understand the disease's patterns and develop targeted prevention and treatment strategies in vulnerable populations.

The study underscores the importance of continued surveillance and research into demographic and regional trends to guide the development of tailored prevention, early detection, and treatment strategies. Furthermore, future research should aim to explore the influence of lifestyle, immune function, and viral dynamics on KS progression and identify risk factors specific to vulnerable groups. Efforts to improve healthcare access, promote early screening, and optimize treatment protocols for high-risk populations remain critical to reducing the burden of KS in the United States. Addressing these gaps can enhance outcomes for patients and contribute to narrowing racial and geographic disparities in cancer care.
